# Candidate single nucleotide polymorphisms of irritable bowel syndrome: a systemic review and meta-analysis

**DOI:** 10.1186/s12876-019-1084-z

**Published:** 2019-10-15

**Authors:** Shiwei Zhu, Ben Wang, Qiong Jia, Liping Duan

**Affiliations:** Department of Gastroenterology, Peking University Third Hospital, No.49 North Garden Rd., Haidian District, Beijing, 100191 China

**Keywords:** Genetic risk, Irritable bowel syndrome, Single nucleotide polymorphisms, TNFSF15 IL10

## Abstract

**Background:**

Genetic factors increase the risk of irritable bowel syndrome (IBS). Analysis of single nucleotide polymorphisms (SNPs) has been used in IBS patients, but the findings are inconsistent. The goal of this review was to synthesize all the published SNPs studies of IBS through meta-analysis to objectively evaluate the relevance of SNPs to IBS risks.

**Methods:**

IBS - related polymorphisms studies from 2000 to 2018 were searched. Pooled odds ratios with a 95% confidence interval for each SNP were evaluated through five genetic models. Ethnicity, ROME criteria and IBS subtypes were defined for subgroup analyze.

**Results:**

Ten relevant genes were evaluated. SNPs rs4263839 and rs6478108 of *TNFSF15* associated with an increased risk of IBS; *IL6* rs1800795 increased the risk for Caucasian IBS patients which diagnosed by Rome III criteria; and *IL23R* rs11465804 increased the risk for IBS-C patients. *IL10* rs1800896 GG genotype associated with a decreased risk of IBS. No evidence supported the association of *GNβ3* rs5443, *TNFα* rs1800629, and *IL10* rs1800871 to IBS in this study.

**Conclusions:**

This meta-analysis presents an in-depth overview for IBS SNPs analysis. It was confirmed that polymorphisms of *TNFSF15* associated with increased IBS risk, while *IL10* rs1800896 associated with decreased IBS risk. It might offer some insights into polymorphisms of inflammation factors which might affect IBS susceptibility. Moreover, the analysis also emphasizes the importance of diagnostic criteria and phenotype homogeneity in IBS genetic studies.

## Background

Irritable bowel syndrome (IBS) is a predominant and common chronic gastrointestinal (GI) disorder presenting with recurrent abdominal pain accompanied with altered bowel habits. IBS has been a continually increasing trend worldwide, especially in developing countries. It leads to negative effects on the quality of life and the work efficiency of affected patients. According to the Rome IV criteria, IBS is categorized into four subtypes [1], diarrhea predominant IBS (IBS-D), constipation predominant IBS (IBS-C), mixture of diarrhea and constipation IBS (IBS-M) and un-subtyped IBS (IBS-U).

Genetic, environmental and psychological factors, which may result in “brain-gut-axis” dysfunction [2], increase the risk of IBS. In addition, the consequential pathophysiological mechanisms [3] such as changes in gastrointestinal motility, visceral hypersensitivity, increasing mucosal permeability, immune activation and gut microbiota dysbiosis, are evaluated in many researches. Due to the multifactorial origin and the elusive etiology of IBS, there is no consensus on diagnostic biomarkers/methods or curative therapy it.

In early 2000, twins [4] and family [5, 6] studies demonstrated a more heritable component to IBS. The associations of IBS and its risk gene polymorphisms have been ascertained by many researchers. Single nucleotide polymorphisms (SNPs) represent the most widespread type of sequence variations in genomes. It is known to be valuable genetic markers, because it may reveal the evolutionary history and common genetic polymorphisms that explain the hereditary risks for common diseases such as inflammation bowel disease (IBD) [7, 8]. Case-control studies have examined the possible role of different SNPs in patients with IBS, such as serotonin transporter protein (SERT) [9], Catechol-O-methyltransferase (COMT) [10], β3 subunit of G-protein (GNβ3) [11], voltage-gated mechanosensitive Na(+) channel NaV1.5 (SCN5A) [12], and tumor necrosis factor (TNF)-α [13]. Some meta-analysis previously were conducted and researchers attempted to extract commonalities as well. Owing to unclear or mixed ethnicity, patients’ population changes, updating of Rome diagnostic criteria and usage of different genetic models, conclusion of association for SNPs and IBS have still been inconsistent over time.

Therefore, this systematic review aimed to synthesize and updated previous SNPs studies through meta-analysis, in order to produce an in-depth analysis of genetic SNPs with IBS from a more detailed perspective.

## Methods

### Search strategy and study selection

Studies of irritable bowel syndrome and its associated genetic polymorphisms were identified by systematically searching from the following databases: PubMed, Web of Science, EMBASE, Cochrane Clinical Trials Database, Medline and Chinese database Chinese National Knowledge Infrastructure. Searching terms of medical subject headings (MeSH) included ‘irritable bowel syndrome, IBS’ combined with ‘polymorphism, genetic polymorphism, single nucleotide polymorphisms, SNPs’. Studies were concerned in the period of 2000.01–2018.01 and the search was not limited by language or publication status. Potentially relevant articles were screened by at least 2 independent reviewers, and disagreements were resolved by discussion or input from a third reviewer if required.

### Inclusion criteria and quality assessment

All candidate studies were included if they met all the inclusion criteria as follow: (i) Case-control studies with subjects’ information, available allele frequency and no consanguinity between the case and control groups. (ii) Explicit ethnicity such as Caucasian or Mongolian. (iii) IBS diagnosis based on clinical examination and specific diagnostic criteria such as Rome I-III. (iv) Allele frequency meets Hardy-Weinberg equilibrium in healthy controls. (v) Largest sample size was included in reused data. Newcastle-Ottawa Quality Assessment Scale (NOS) scored as quality assessment in all studies. To confirm the test effect, SNPs that had been reported in less than 3 studies were excluded in this meta-analysis.

### Data extraction

Two investigators independently extracted data from the identified publications, including the first author’s name, year of publication, source of publication, IBS diagnostic criteria, DNA extraction and genotyping method, numbers and source of patients and controls, genotype frequency, and allele frequency. Discrepancies in data extraction were resolved by repeating the study review and discussing the results. The corresponding author was contacted, and genotype frequencies were requested when missing from the studies.

### Statistical analysis

Hardy-Weinberg equilibrium (HWE) analysis of the controls was performed using the Chi-square test. To determine the overall gene effect, five genetic models including allele (AM), dominant (DM), recessive (RM), homozygous (HoM) and heterozygous (HeM) models were used to evaluate the allele and genotype risks for IBS [14, 15]. Relative risks of IBS were estimated according to odds ratios (ORs) with 95% confidence intervals (CIs). The heterogeneity among studies was assessed using the Cochran Q test [16]. The inconsistency index I^2^ was also calculated to quantify heterogeneity. A fixed-effects model was used to pool the results if the result of the heterogeneity test was not significant (*P* > 0.05) or I^2^ < 50%); otherwise, a random-effect model was selected. Sensitive test was conducted to determine the source of heterogeneity. Publication bias was examined by using the Begg’s test only if analyzed studies were more than five [17]. Subgroups of ethnicity, diagnostic criteria and IBS subtypes were conducted in each SNP. All statistical tests were two-tailed, and the level of significance was set at *P* < 0.05. STATA version 13 (Stata Corporation, College Station, TX, United States) was used for all analysis.

## Results

### Study selection and characteristics analysis

**Fig. 1:**
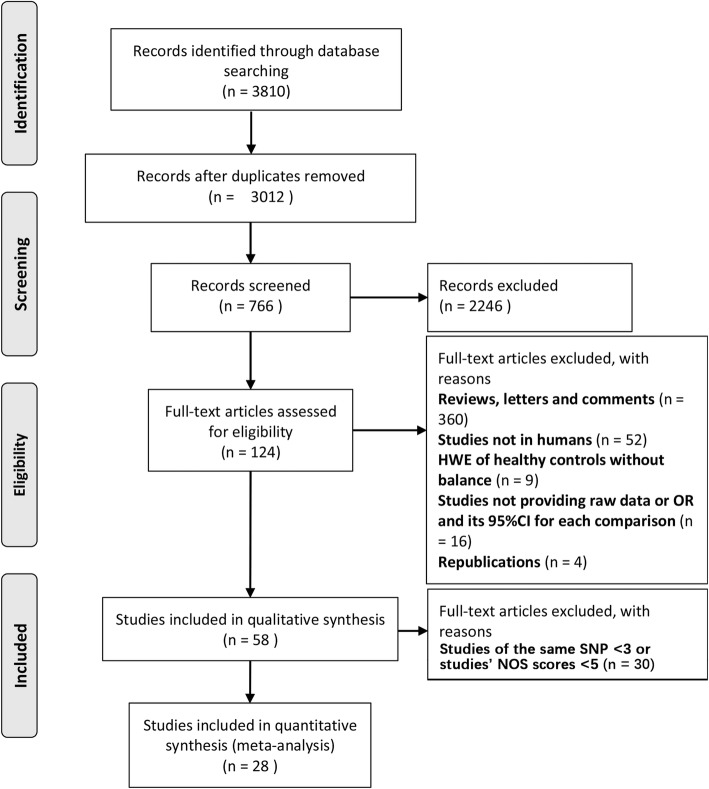
Flow diagram of study selection for the meta-analysis

From the databases, 3810 potentially relevant publications were identified. Figure 1 shows the flow diagram of this study. After screening and inclusion, 66 SNPs were identified in IBS patients (see Additional file 1: Table S1). Finally, 10 different SNPs from 28 studies were determined; references for these studies are provided in Additional file 1: Table S1. The identified SNPs focus on neurotransmitter system (*SLC6A4 5-HTTLPR*, *COMT rs4680* and *GNβ3 rs5443*) and the inflammation system (*TNFα rs1800629, IL10 rs1800896, IL10 rs1800871, IL6 rs1800795, IL23R rs11465804, TNFSF15 rs4263839* and *TNFSF15 rs6478108*). Most of the studies reported that the DNA was extracted from blood, except for 2 studies that had DNA extracted from buccal epithelial cells and sputum. SNPs were assayed through PCR. The characteristics of these studies are shown in Additional file 1: Table S2. Rome III criteria was used in half of the studies (50% Rome III, 46.6% Rome II and 0.04% Rome I). Multiple comparisons of identified SNPs through five genetic models is summarized in Table 1.

**Table 1:** Summary of results of all polymorphisms for five genetic models

Gene rs.	Gene Model	*p1* value^a^	OR (96% CI)^b^	*p2* value^c^	Analysis model
SLC6A4 5-HTTLPR	AM: s vs. l	0.008	1.169 (0.908, 1.505)	0.226	REM
	DM: ls + ss vs. ll	0.013	0.967 (0.689, 1.358)	0.848	REM
	RM: ss vs. ll + ls	0.008	1.169 (0.908, 1.505)	0.226	REM
	HoM: ss vs. ll	0.004	1.114 (0.724, 1.714)	0.623	REM
	HeM: ls vs. ll	0	2.312 (1.084, 4.931)	0.03	REM
COMT rs4680	AM: A vs. G	0.07	1.011 (0.843, 1.212)	0.91	FEM
	DM: GA + AA vs. GG	0.311	0.827 (0.632, 1.082)	0.167	FEM
	RM: AA vs. GG + GA	0.044	1.461 (0.730, 2.924)	0.284	REM
	HoM: AA vs. GG	0.018	1.291 (0.522, 3.189)	0.581	REM
	HeM: GA vs.GG	0.141	0.673 (0.5, 0.907)	0.009	FEM
TNFα rs1800629	AM: A vs. G	0.301	0.95 (0.831, 1.086)	0.453	FEM
	DM: GA + AA vs. GG	0.315	0.866 (0.576, 1.303)	0.49	FEM
	RM: AA vs. GG + GA	0.107	0.895 (0.587, 1.364)	0.606	FEM
	HoM: AA vs. GG	0.415	0.854 (0.565, 1.290)	0.453	FEM
	HeM: GA vs.GG	0.096	0.954 (0.856, 1.111)	0.543	FEM
IL10 rs1800896	AM: G vs. A	0.774	0.935 (0.826, 1.508)	0.286	FEM
	DM: GA + GG vs. AA	0.842	1.024 (0.842, 1.245)	0.815	FEM
	RM: GG vs. AA + GA	0.321	0.806 (0.655, 0.992)	0.042	FEM
	HoM: GG vs. AA	0.719	0.855 (0.661, 1.105)	0.23	FEM
	HeM: GA vs. AA	0.659	1.113 (0.906, 1.367)	0.036	FEM
IL10 rs1800871	AM: C vs. T	0.825	0.944 (0.764, 1.167)	0.596	FEM
	DM: TC + CC vs. TT	0.6	1.022 (0.766, 1.345)	0.878	FEM
	RM: CC vs. TT + TC	0.647	0.701 (0.434, 1.133)	0.147	FEM
	HoM: CC vs. TT	0.596	0.743 (0.444, 1.244)	0.259	FEM
	HeM: TC vs.TT	0.496	1.092 (0.818, 1.456)	0.551	FEM
IL6 rs1800795	AM: G vs. C	0.039	1.144 (0.810, 1.373)	0.249	REM
	DM: CG + GG vs. CC	0.292	1.092 (0.888, 1.346)	0.4	FEM
	RM: GG vs. CC + CG	0	1.373 (0.858, 2.198)	0.186	REM
	HoM: GG vs. CC	0.657	1.099 (0.872, 1.387)	0.657	FEM
	HeM: CG vs.CC	0.038	0.905 (0.566, 1.449)	0.679	REM
IL23R rs11465804	AM: G vs. T	0.005	1.266 (0.813, 1.971)	0.296	REM
	DM: TG + GG vs. TT	0.004	1.265 (0.789, 2.029)	0.329	REM
	RM: GG vs. TT + TG	0.902	1.21 (0.460, 3.183)	0.699	FEM
	HoM: GG vs. TT	0.004	1.244 (0.770, 2.009)	0.372	REM
	HeM: TG vs.TT	0.896	1.209 (0.983, 1.487)	0.072	FEM
TNFSF15 rs4263839	AM: G vs. A	0	5.139 (3.859, 6.844)	0	REM
	DM: GA + GG vs. AA	0.012	6.527 (4.616, 9.229)	0	REM
	RM: GG vs. AA+GA	0	2.802 (0.951, 8.261)	0.062	REM
	HoM: GG vs. AA	0.557	19.127 (15.395, 23.765)	0	FEM
	HeM: GA vs. AA	0	9.361 (4.702, 18.637)	0	REM
TNFSF15 rs6478108	AM: T vs. C	0.288	1.143 (1.016, 1.287)	0.026	FEM
	DM: CT + TT vs. CC	0.335	1.235 (0.964, 1.581)	0.094	FEM
	RM: TT vs. CC + CT	0.23	1.171 (0.997, 1.374)	0.054	FEM
	HoM: TT vs. CC	0.287	1.306 (1.005, 1.697)	0.045	FEM
	HeM: CT vs.CC	0.326	1.17 (0.902, 1.519)	0.237	FEM
GNβ3 rs5443	AM: T vs. C	0.013	1.167 (0.825, 1.651)	0.383	REM
	DM: CT + TT vs. CC	0.025	1.196 (0.762, 1.877)	0.437	REM
	RM: TT vs. CC + CT	0.227	1.273 (0.811, 1.998)	0.295	FEM
	HoM: TT vs. CC	0.037	1.394 (0.701, 2.772)	0.344	REM
	HeM: CT vs.CC	0.088	1.166 (0.776, 1.753)	0.459	FEM

a Cochran Q test;b Odds ratio (95% confidence interval); c Mante-Haenszel test; *AM* Allele models, *DM* Dominant models, *RM* Recessive models, *HoM* Homozygous models, *HeM* Heterozygous models, *REM* Random effect model, *FEM* Fixed effect model

### SLC6A4 5-HTTLPR and IBS risk

**Fig. 2:**
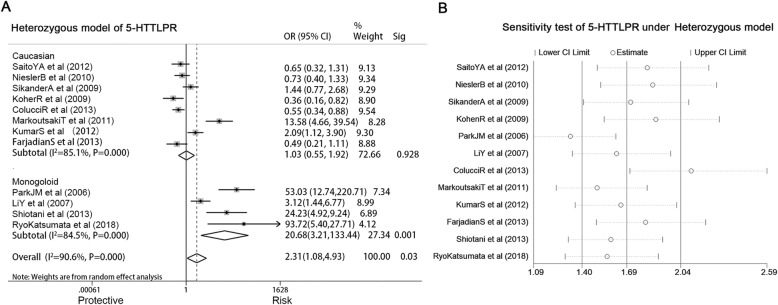
**a**, Ethnicity subgroup analysis showed in forest plot of the associations between the 5-HTTLPR polymorphism *ls* genotype and IBS risk in the heterozygous model; **b**, Sensitivity test of studies reported the association between *SLC6A4 5-HTTLPR* polymorphism and IBS risk in the heterozygous model (p, significance of Cochran Q test; Sig, significance of Mante-Haenszel test)

Twelve studies involving 1834 IBS subjects and 1941 controls were analyzed to determine the association of the *SLC6A4 5-HTTLPR* and IBS risk (Table 1). Genotype *ls* presented an increased risk for IBS development in HeM (*ls* vs. *ll,* OR = 2.312, 95% CI: 1.084–4.931, *P =* 0.03) (Fig. 2a). Heterogeneity for included studies is significant (*P <* 0.05). A sensitivity analysis, after excluding studies in turn, indicated that the associations remained (Fig. 2b). Begg’s test suggested no publication bias (*P* = 0.064). Thus, subgroup analysis based on ethnicity or diagnostic criteria was performed. Figure 2a shows that polymorphism was significantly correlated with IBS risk in Mongoloid population (OR = 20.68, 95% CI: 3.21–133.44, *P =* 0.001), but there was no association in Caucasian populations. No significant association was found in diagnostic criteria subgroups. Further, IBS subtypes (IBS-A, IBS-C and IBS-D) were analyzed but no association was found.

### *COMT* rs4680 and IBS risk

**Fig. 3:**
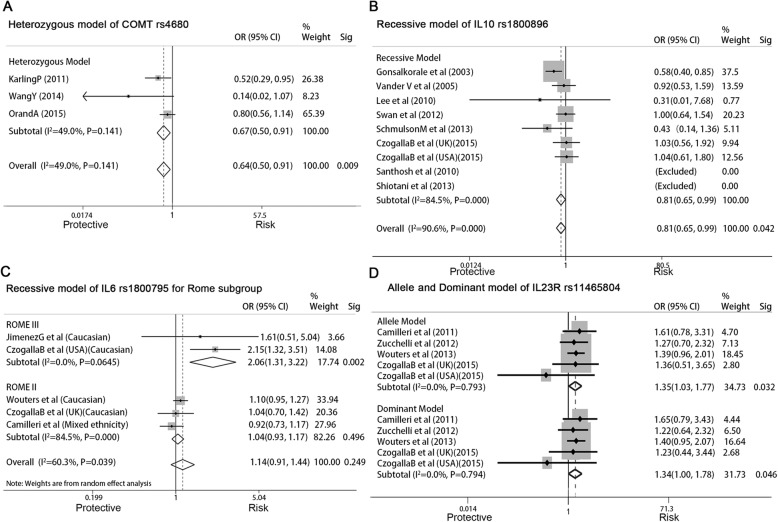
**a**, Forest plot of the associations between the *COMT* rs4680 GA genotype and IBS risk in heterozygous model; **b**, Forest plot of the association between the *IL10* rs1800896 GG genotype and IBS risk in recessive model; **c**, Diagnostic criteria subgroup showed in forest plot of the association between the *IL6* rs1800795 G allele and IBS risk in recessive model; **d**, Forest plot of the association between the *IL23R* rs11465804 polymorphism and IBS-C risk in allele and dominant models (p, significance of Cochran Q test; Sig, significance of Mante-Haenszel test)

Three studies involving 414 IBS patients and 1363 controls were analyzed for the association of *COMT* rs4680 (G > A) and IBS risk (Table 1). GA genotype presented a decreased risk for IBS in the HeM (GA vs. GG, OR = 0.673, 95% CI: 0.5–0.907, *P =* 0.009) (Fig. 3a). Included studies were with a good homogeneity (I^2^ = 49%, *P* = 0.141). Subgroups analyses were conducted but no associations were found.

### *IL10* rs1800896 and IBS risk

Seven studies involving 955 IBS patients and 779 controls were analyzed for the association of *IL10* rs1800896 (A > G) and IBS risk (Table 1). GG genotype presented a decreased risk of IBS in the RM (GG vs. GA+AA, OR = 0.806, 95% CI: 0.655–0.992, *P =* 0.042) (Fig. 3b). No significant heterogeneity (I^2^ = 14.3%, *P* = 0.321) was found. Further subgroup analysis was used for ethnicity and diagnostic criteria, but no additional associations were found.

### *IL6 rs1800795* and IBS risk

There were four studies involving 1641 IBS patients and 1058 controls, which were analyzed for the association of *IL6* rs1800795 (C > G) and IBS risk (Table 1). The data showed no association of allele or genotype with IBS risk. The AM (G vs. C) was used for subgroup analysis. This finding was interesting because there was no association of the G allele with IBS in the Caucasian subgroup, but in Caucasian subgroups with diagnostic Rome III criteria (Fig. 3c), the *IL6* rs1800795 G allele significantly increased the risk for IBS (OR = 2.057, 95% CI: 1.313–3.225, *P =* 0.002).

### IL23R rs11465804 and IBS risk

There were four studies involving 2068 IBS patients and 1958 controls that analyzed the association of *IL23R* rs11465804 (T > G) and IBS risk (Table 1). The data showed no association of the polymorphism with IBS risk in any of the models. In subgroup analysis of IBS subtype, *IL23R* rs11465804 increased the risk for IBS-C both in AM (G vs. T, OR = 1.346, 95% CI: 1.025–1.767, *P =* 0.032) and DM (TG + GG vs. TT, OR = 1.338, 95% CI: 1.005–1.781, *P =* 0.046) (Fig. 3d). No association was found in IBS-D patients or other subgroup.

### TNFSF15 rs4263839 and IBS risk

**Fig. 4:**
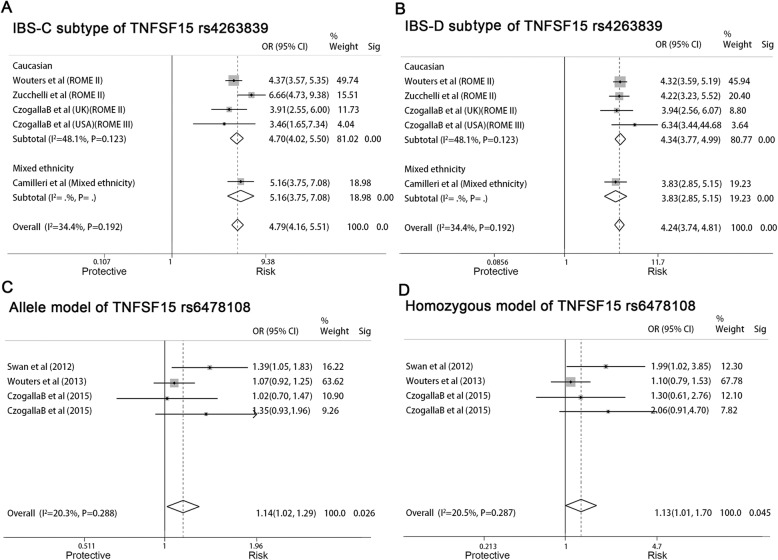
**a**, **b**, Forest plot of the associations between the *TNFSF15* rs4263839 polymorphism and IBS-C (**a**), IBS-D (**b**) risk in allele model; **c**, **d**, Forest plot of the association between the *TNFSF15* rs6478108 polymorphism and IBS risk in allele (**c**) and recessive (**d**) model (p, significance of Cochran Q test; Sig, significance of Mante-Haenszel test)

Four studies involving 2068 IBS patients and 1959 controls analyzed the association of *TNFSF15* rs4263839 (A > G) and IBS risk (Table 1). A significantly positive association between*TNFSF15* rs4263839 polymorphism and IBS development was found in AM (G vs. A, OR = 5.139, 95% CI: 3.859–6.844, *P* < 0.01), DM (GA + GG vs. AA, OR = 6.527, 95% CI: 4.616–9.229, *P* < 0.01), HoM (GG vs. AA, OR = 19.127, 95% CI: 15.395–23.765, *P* < 0.01) and HeM (GA vs. AA models, OR = 9.361, 95% CI: 4.702–18.637, *P* < 0.01). AM was used for subgroup analysis. As for IBS subtype (Fig. 4a and b), the G allele increased the risks for both IBS-C (OR = 4.79, 95% CI: 4.16–5.51, *P* < 0.01) and IBS-D (OR = 4.24, 95% CI: 3.74–4.81, *P* < 0.01). Moreover, subgroup analysis of Caucasian (Fig. 4a and b) also supported the results.

### TNFSF15 rs6478108 and IBS risk

There were three studies involving 1527 IBS patients and 1008 controls that analyzed the association of *TNFSF15* rs6478108 (C > T) and IBS risk (Table 1). Polymorphism increases the risk of IBS in AM (T vs. C, OR = 1.043, 95% CI: 1.016–1.287, *P =* 0.026) and HoM (TT vs. CC models, OR = 1.306, 95% CI: 1.005–1.697, *P =* 0.045) (Fig. 4c and d) accompany with good homogeneity (AM: I^2^ = 20.3%, *P* = 0.288; HoM: I^2^ = 20.5%, *P* = 0.287). Because all the subjects participating in these studies were Caucasian, only subgroup analysis of diagnostic criteria was performed, but the results suggested no correlations.

### SNPs had no association with IBS risk

Eight studies involving 1868 IBS patients and 1462 controls were analyzed for the association of *TNFα* rs1800629 (G > A), four studies involving 470 IBS patients and 485 controls were analyzed for the association of *IL10* rs1800871 (T > C), four studies involving 724 IBS patients and 839 controls that analyzed the association of *GNβ3* rs5443 (C > T) and IBS risk (Table 1). Five genetic models were used for analysis, but no association of polymorphism with IBS risk was found in any of the models. In addition, the AM was used for subgroup analysis, there was no association in the subgroup analysis.

## Discussion

As a multi-pathogenesis disease, the genetic risk [6, 18] of IBS have been demonstrated in many studies. More than 65 candidate genes have been reported for IBS. Many new IBS associated SNPs was found through different strategies, for example, the genome-wide association studies (GWAS) [19–21]. However, consensus of the major IBS risk genes has been hard to reach. F. Bonfiglio et al. [22] carried out a GWAS meta-analysis of patients with IBS, they found SNPs in regulation of ion channel activity such as *SCN5A* and *SI* as the most plausible pathway affecting IBS. However, GWAS origin risk genes have not been successfully replicated in independent studies. Those IBS risk SNPs are mainly located in introns or UTR regions, which complicating the explanation of the gene functions to IBS pathygenesis. Moreover, most of the IBS GWAS analysis are population-based rather than identified as IBS cohort-based, which may cause variations. With the development of techniques, more newly detected SNPs were found related with the development of IBS in case-control studies. For example, SNPs of calcium-sensing receptor polymorphism (CaSR) [23] rs1801725 and adrenergic receptor (ADR) [24–26]. Nevertheless, there is no overview of all IBS-associated polymorphisms. Thus, this systemic review synthesized all the published SNPs studies of IBS through a strict meta-analysis, with the goal of objectively determining the relevance of genetic SNPs with IBS.

In this study, 10 relevant SNPs from 28 studies were evaluated. Many other SNPs which reported in less than three studies or had unclear allele frequency in articles were not included, even if they were latest reported. A study by Czogalla et al. [10] was included because Czogalla and their colleagues utilized two independent case-control cohorts (UK and USA cohorts) and identified risk SNPs separately. Thus, we considered these data as two cohorts in our analysis. Allele model and other four genotype model (DM, RM, HeM and HoM) were used to give an exhaustive analysis of the association. Except for ethnicity subgroup, diagnostic criteria and IBS subtype were also defined as another two subgroups which might assist to further analysis.

Cytokine gene polymorphisms are important because they might be associated with changes in cytokine profiles. It represent immune system dysregulation in IBS development. Among all the SNPs, *TNFSF15* rs4263839 and *TNFSF15* rs6478108 increased the risk of IBS. *TNFSF15* encodes for TL1A, which is a tumor necrosis factor superfamily member expressed in different immune cells. It may trigger an immune response through Th17 cell [27] and play an important role in the development of many autoimmune and inflammatory diseases. Studies have demonstrated a close association between TL1A and IBD. Genetic analysis also confirmed that the *TNFSF15* gene is a race-specific susceptibility gene for IBD [28, 29] and TL1A was up-regulated both in intestinal mucosal T-cells and peripheral blood mononuclear cells of IBD patients [30]. Animal experiments showed that anti-TL1A antibody could reduce intestinal inflammation in chronic colitis [28]. According to the results of this study, *TNFSF15* rs4263839 G allele increasing the risk of IBS. It was found in IBS patients and different IBS subtypes (IBS-C, IBS-D). *TNFSF15* rs6478108 T allele increased IBS risk as well, but no association was found in subgroup analysis. This finding might provide a clue for the overlaps between IBD and IBS, and it might become a treatment target for IBS. For another Th17-associated pathway, IL23R interacts with IL23 to regulate the activity of immune cells and plays an important role in the inflammatory response against infection by bacteria and viruses. *IL23R* rs11465804, which associated with increasing risk of IBD [7], has also been reported in case-control studies and GWAS in patients with IBS. It was hypothesized [31] that *IL23R* gene variants increased the secretion of Th17 in patients, leading to a protective effect. In this study, *IL23R* rs11465804 G allele of IBS-C patients represented a protective effect. However, fewer studies focus on *IL23R* rs11465804, and its function on intestinal motility is unclear which needs further analysis.

IL6 has been reported increasing in the plasma of IBS patients. *IL6* rs1800795 mutation (C > G) is associated with higher plasma concentrations of IL6 during immune activation [32]. Our finding is intriguing, *IL6* rs1800795 G allele doubled the risk of Caucasian IBS patients which diagnosed by Rome III criteria but not Rome II criteria. It might because IBS diagnostic criteria changed greatly from Rome II to Rome III, the later defined different IBS subtypes based on Bristol scale, which purifying IBS patients from other functional gastroenterology diseases. For *IL10* rs1800896, people with GG allele seem to have lower risks developing to IBS. This result is consistent with previous studies [13]. In addition, a few studies confirmed a decreased IL10 level in the serum and intestinal mucosa of IBS patients. Probiotics, such as *Bifidobacterium* and *Lactococcus,* can regulate IL10 level to reduce mucosal inflammation [32–34].

Serotonin is an important neurotransmitter both in the CNS and GI tract. It is reuptaken by SERT which encoded by the *SLC6A4* gene to regulate serotonin concentration. Case–control studies on *SLC6A4 5-HTTLPR* were conducted to verify this hypothesis. Some studies demonstrated a positive association while others failed to confirm that [35]. Mohammed YA et al. reported *s* allele of *SLC6A4 5-HTTLPR* reduced the risk of IBS in Asian population, while another meta-analysis [9] found *l* allele uniquely associated with increased IBS-C risk. Data in this meta-analysis only represented that *ls* genotype of *SLC6A4 5-HTTLPR* associated with increased risk of IBS in Mongoloid ethnicity. No association was found in other genetic models and further subgroup analysis. *SLC6A4 5-HTTLPR* with a short variation (*s*) has been shown to decrease the activity of SERT which may accelerate intestinal peristalsis. However, studies took for analysis have significant heterogeneity, further analyses are necessary to confirm the results. Though, serotonin plays a key role in intestinal motility, sensitivity and endocrine systems, due to its wide distribution and nonspecific effect, serotonin can also be influenced by IBS subtype, ethnicity and many other factors. It is very difficult to support a strong relationship of serotonin-associated polymorphisms with IBS. Based on the studies above and our results, it is understandable that serotonin has been the earliest treatment target of IBS but with a little application. GA genotype of *COMT* rs4680 was associated with decreased IBS risk. COMT is an enzyme involved in the degradation of catecholamine neurotransmitters. *COMT* rs4680 leads to the substitution of valine (Val) by methionine (Met), which decreases the enzyme activity and is associated with a lower pain sensitivity threshold [24]. However, our result is not consistent with previous studies [36, 37]. One reason might be the limited studies with mixed ethnicity - only three studies were analyzed, but Mongoloid, Caucasian, and other ethnicities were all included. Another reason might be the different DNA sources. For example, Orand et al. [24] extracted DNA from saliva. No evidence for a contribution of *GNβ3* rs5443, *TNFα* rs1800629, and *IL10* rs1800871 to IBS was found in this study, which is consistent with previous researches [10, 11, 38].

Some limitations to this meta-analysis require careful consideration. First, due to the rigorous filtering criteria, limited data were available. Hence, other factors such as SNPs detecting methods, environmental factors and the source of healthy controls for comparison, which may also affect susceptibility to IBS, were not accounted for in the present study. Second, allele and genotype effect on IBS risk were both analyzed but no best genetic model was determined. Differ from those monogenous hereditary diseases, the pathogenesis of IBS is the result of the combination of both environmental and genetic factors. It’s hard to tell whether someone will develop IBS by having a specific allele mutation. Moreover, multiple comparisons through different genetic models can increase the probability of false-positive outcome as well.

## Conclusions

In this review, it was confirmed that *TNFSF15* rs4263839 and *TNFSF15* rs6478108 associated with increased IBS risk, while *IL10* rs1800896 GG genotype associated with decreased IBS risk. Diagnostic criteria changes had influence on the association between *IL6* rs1800795 and IBS risk. And *IL23R* rs11465804 might become a new target for IBS-C developing. According to these findings, it might offer some insights into gene functions affecting IBS susceptibility and some clues in IBS genetic analysis.

## Supplementary information

**Additional file 1:** **Table S1.** Summary of studied according to SNP: this table summarized all the SNPs which had been reported in IBS and shows its reference. **Table S2.** Study characteristics analysis: this table shows the most important information for the studies which take into analysis in this SMRA.

## Data Availability

The datasets used and/or analyzed during the current study are available from the corresponding author on reasonable request.

## Abbreviations

CI: Confidence interval
COMT: Catechol-O-methyltransferase
GI: Gastrointestinal
GNβ3: β3 subunit of G-protein
GWAS: Genome-wide association studies
HWE: Hardy-Weinberg equilibrium
IBD: Inflammation bowel disease
IBS: Irritable bowel syndrome
IBS-C: Constipation predominant IBS
IBS-D: Diarrhea predominant IBS
IBS-M: Mixture of diarrhea and constipation IBS
IBS-U: Unsubtyped IBS
IL: Interleukin
MeSH: Medical subject headings
OR: Odds ratios
SERT: Serotonin transporter
SNPs: Single nucleotide polymorphisms
TNF: Tumor necrosis factor
