# Supplementary material for: Candidate single nucleotide polymorphisms of irritable bowel syndrome: a systemic review and meta-analysis
Source: BMC Gastroenterol. 2019 Oct 15;19:165. doi: 10.1186/s12876-019-1084-z (PMC6792237; doi:10.1186/s12876-019-1084-z)
Supplement: Supplementary file 1 — Additional file 1: Table S1. Summary of studied according to SNP: this table summarized all the SNPs which had been reported in IBS and shows its reference. Table S2. Study characteristics analysis: this table shows the most important information for the studies which take into analysis in this SMRA. [file 12876_2019_1084_MOESM1_ESM.docx]

| **Additional Table1. Summary of studied according to SNP** | | | | | |
| --- | --- | --- | --- | --- | --- |
| **SNPs** | **Count of study** | **SNPs** | **Count of study** | **SNPs** | **Count of study** |
| **SLC6A4 5-HTTLPR**[1-12] | **14** | TPH1 rs211105[13, 14] | 2 | FAAH rs324420[15] | 1 |
| **TNFα rs1800629**[16-23] | **8** | ADRA1D rs1556832[24] | 1 | HTR3A rs1062613[25] | 1 |
| **IL10 rs1800896**[16, 18-20, 22, 23, 26, 27] | **7** | ADRA1D rs946188[24] | 1 | HTR3E rs56109847[28] | 1 |
| **IL23R rs11465804**[21, 23, 29, 30] | **4** | ADRA2A rs1800544[31] | 1 | IFNγ rs62559044[18] | 1 |
| **GNβ3 rs5443**[31-34] | **4** | ADRAβ2 rs1042713[24] | 1 | IL10 rs3021097[35] | 1 |
| **COMT rs4680**[24, 34, 36] | **3** | ADRAβ2 rs1042717[24] | 1 | IL1α rs1800587[35] | 1 |
| **IL10 rs1800871**[16, 18, 23, 27] | **4** | ADRAβ2 rs1432622[24] | 1 | IL1β rs1143634[35] | 1 |
| **IL6 rs1800795**[17, 21, 23, 29] | **4** | ADRAβ2 rs2400707[24] | 1 | IL2 rs2069762[37] | 1 |
| **TNFSF15 rs4263839**[21, 23, 29, 30] | **4** | ANKK1 rs1800497[24] | 1 | IL6 rs1800797[35] | 1 |
| **TNFSF15 rs6478108**[19, 21, 23] | **3** | COMT rs174697[24] | 1 | IL6 rs2069861[23] | 1 |
| HT2A rs6311[38, 39] | 2 | COMT rs6269[24] | 1 | IL8 rs2227307[23] | 1 |
| HT2A rs6313[38, 39] | 2 | CRH rs28364015[40] | 1 | SLC6A4 rs1042173[41] | 1 |
| IL10 rs1800872[18, 35] | 2 | CRHR1 rs110402[42] | 1 | SLC6A4 rs2020936[41] | 1 |
| IL1R1 rs2234650[23, 35] | 2 | CRHR1 rs242924[42] | 1 | SLC6A4 rs25531[43] | 1 |
| IL4 rs2070874[21, 23] | 2 | CRHR2 rs2190242[44] | 1 | TGFβ rs1800470[16] | 1 |
| IL4 rs2243250[21, 23] | 2 | CRHR2 rs2240403[44] | 1 | TGFβ rs1800471[16] | 1 |
| IL8 rs2227306[23, 27] | 2 | CRHR2 rs2267710[44] | 1 | TPH1 rs4537731[45] | 1 |
| IL8 rs4073[23, 27] | 2 | CRHR2 rs2284217[44] | 1 | TPH1 rs684302[45] | 1 |
| SLC6A4 rs2066713[46, 47] | 2 | CRHR2 rs2284220[44] | 1 | TPH1 rs7130929[48] | 1 |
| TNFSF15 rs6478109[19, 23] | 2 | CRHR2 rs3779250[44] | 1 | TPH2 rs4570625[45] | 1 |
| TNFSF15 rs7848647[19, 23] | 2 | CRHR2 rs4722999[44] | 1 | TRPV1 945G>C[31] | 1 |
| TNFα rs361525[17, 23, 43] | 2 | DRD3 rs6280[49] | 1 | SCN5A rs7430407[50] | 1 |

## References:

1. Colucci R, Gambaccini D, Ghisu N, Rossi G, Costa F, Tuccori M, De Bortoli N, Fornai M, Antonioli L, Ricchiuti A *et al*: **Influence of the serotonin transporter 5HTTLPR polymorphism on symptom severity in irritable bowel syndrome**. *Plos One* 2013, **8**(2):e54831.

2. Farjadian S, Fakhraei B, Moeini M, Nasiri M, Fattahi MR: **Serotonin transporter gene polymorphisms in Southwestern Iranian patients with irritable bowel syndrome**. *Arab J Gastroenterol* 2013, **14**(2):59-62.

3. Katsumata R, Shiotani A, Murao T, Ishii M, Fujita M, Matsumoto H, Haruma K: **The TPH1 rs211105 gene polymorphism affects abdominal symptoms and quality of life of diarrhea-predominant irritable bowel syndrome**. *J Clin Biochem Nutr* 2018, **62**(3):270-276.

4. Kohen R, Jarrett ME, Cain KC, Jun SE, Navaja GP, Symonds S, Heitkemper MM: **The serotonin transporter polymorphism rs25531 is associated with irritable bowel syndrome**. *Dig Dis Sci* 2009, **54**(12):2663-2670.

5. Kumar S, Ranjan P, Mittal B, Ghoshal UC: **Serotonin transporter gene (SLC6A4) polymorphism in patients with irritable bowel syndrome and healthy controls**. *J Gastrointestin Liver Dis* 2012, **21**(1):31-38.

6. Li Y, Nie Y, Xie J, Tang W, Liang P, Sha W, Yang H, Zhou Y: **The association of serotonin transporter genetic polymorphisms and irritable bowel syndrome and its influence on tegaserod treatment in Chinese patients**. *Dig Dis Sci* 2007, **52**(11):2942-2949.

7. Markoutsaki T, Karantanos T, Gazouli M, Anagnou NP, Ladas SD, Karamanolis DG: **Serotonin transporter and G protein beta 3 subunit gene polymorphisms in Greeks with irritable bowel syndrome**. *Dig Dis Sci* 2011, **56**(11):3276-3280.

8. Niesler B, Kapeller J, Fell C, Atkinson W, Moller D, Fischer C, Whorwell P, Houghton LA: **5-HTTLPR and STin2 polymorphisms in the serotonin transporter gene and irritable bowel syndrome: effect of bowel habit and sex**. *Eur J Gastroenterol Hepatol* 2010, **22**(7):856-861.

9. Park JM, Choi MG, Park JA, Oh JH, Cho YK, Lee IS, Kim SW, Choi KY, Chung IS: **Serotonin transporter gene polymorphism and irritable bowel syndrome**. *Neurogastroenterol Motil* 2006, **18**(11):995-1000.

10. Saito YA, Larson JJ, Atkinson EJ, Ryu E, Almazar AE, Petersen GM, Talley NJ: **The role of 5-HTT LPR and GNbeta3 825C>T polymorphisms and gene-environment interactions in irritable bowel syndrome (IBS)**. *Dig Dis Sci* 2012, **57**(10):2650-2657.

11. Shiotani A, Kusunoki H, Kimura Y, Ishii M, Imamura H, Tarumi K, Manabe N, Kamada T, Hata J, Haruma K: **S100A expression and interleukin-10 polymorphisms are associated with ulcerative colitis and diarrhea predominant irritable bowel syndrome**. *Dig Dis Sci* 2013, **58**(8):2314-2323.

12. Sikander A, Rana SV, Sinha SK, Prasad KK, Arora SK, Sharma SK, Singh K: **Serotonin transporter promoter variant: Analysis in Indian IBS patients and control population**. *J Clin Gastroenterol* 2009, **43**(10):957-961.

13. Katsumata R, Shiotani A, Murao T, Ishii M, Fujita M, Matsumoto H, Haruma K: **The TPH1 rs211105 gene polymorphism affects abdominal symptoms and quality of life of diarrhea-predominant irritable bowel syndrome**. *J Clin Biochem Nutr* 2018, **62**(3):270-276.

14. Jun SE, Kohen R, Cain KC, Jarrett ME, Heitkemper MM: **TPH gene polymorphisms are associated with disease perception and quality of life in women with irritable bowel syndrome**. *Biol Res Nurs* 2014, **16**(1):95-104.

15. Jiang Y, Nie Y, Li Y, Zhang L: **Association of cannabinoid type 1 receptor and fatty acid amide hydrolase genetic polymorphisms in Chinese patients with irritable bowel syndrome**. *J Gastroen Hepatol* 2014, **29**(6):1186-1191.

16. Barkhordari E, Rezaei N, Mahmoudi M, Larki P, Ahmadi-Ashtiani HR, Ansaripour B, Alighardashi M, Bashashati M, Amirzargar AA, Ebrahimi-Daryani N: **T-helper 1, T-helper 2, and T-regulatory cytokines gene polymorphisms in irritable bowel syndrome**. *Inflammation* 2010, **33**(5):281-286.

17. Jimenez-Gonzalez DE, Martinez-Flores WA, Reyes-Gordillo J, Ramirez-Miranda ME, Arroyo-Escalante S, Romero-Valdovinos M, Stark D, Souza-Saldivar V, Martinez-Hernandez F, Flisser A *et al*: **Blastocystis infection is associated with irritable bowel syndrome in a Mexican patient population**. *Parasitol Res* 2012, **110**(3):1269-1275.

18. Santhosh S, Dutta AK, Samuel P, Joseph AJ, Ashok KJ, Kurian G: **Cytokine gene polymorphisms in irritable bowel syndrome in Indian population--a pilot case control study**. *Trop Gastroenterol* 2010, **31**(1):30-33.

19. Swan C, Duroudier NP, Campbell E, Zaitoun A, Hastings M, Dukes GE, Cox J, Kelly FM, Wilde J, Lennon MG *et al*: **Identifying and testing candidate genetic polymorphisms in the irritable bowel syndrome (IBS): association with TNFSF15 and TNFalpha**. *Gut* 2013, **62**(7):985-994.

20. Van der Veek PP, van den Berg M, de Kroon YE, Verspaget HW, Masclee AA: **Role of tumor necrosis factor-alpha and interleukin-10 gene polymorphisms in irritable bowel syndrome**. *Am J Gastroenterol* 2005, **100**(11):2510-2516.

21. Wouters MM, Lambrechts D, Knapp M, Cleynen I, Whorwell P, Agreus L, Dlugosz A, Schmidt PT, Halfvarson J, Simren M *et al*: **Genetic variants in CDC42 and NXPH1 as susceptibility factors for constipation and diarrhoea predominant irritable bowel syndrome**. *Gut* 2014, **63**(7):1103-1111.

22. Lee HJ, Lee SY, Choi JE, Kim JH, Sung IK, Park HS, Jin CJ: **G protein beta3 subunit, interleukin-10, and tumor necrosis factor-alpha gene polymorphisms in Koreans with irritable bowel syndrome**. *Neurogastroenterol Motil* 2010, **22**(7):758-763.

23. Czogalla B, Schmitteckert S, Houghton LA, Sayuk GS, Camilleri M, Olivo-Diaz A, Spiller R, Wouters MM, Boeckxstaens G, Bermejo JL *et al*: **A meta-analysis of immunogenetic Case-Control Association Studies in irritable bowel syndrome**. *Neurogastroenterol Motil* 2015, **27**(5):717-727.

24. Orand A, Gupta A, Shih W, Presson AP, Hammer C, Niesler B, Heendeniya N, Mayer EA, Chang L: **Catecholaminergic Gene Polymorphisms Are Associated with GI Symptoms and Morphological Brain Changes in Irritable Bowel Syndrome**. *Plos One* 2015, **10**(8):e135910.

25. Kilpatrick LA, Labus JS, Coveleskie K, Hammer C, Rappold G, Tillisch K, Bueller JA, Suyenobu B, Jarcho JM, McRoberts JA *et al*: **The HTR3A polymorphism c. -42C>T is associated with amygdala responsiveness in patients with irritable bowel syndrome**. *Gastroenterology* 2011, **140**(7):1943-1951.

26. Schmulson M, Pulido-London D, Rodriguez O, Morales-Rochlin N, Martinez-Garcia R, Gutierrez-Ruiz MC, Lopez-Alvarenga JC, Gutierrez-Reyes G: **IL-10 and TNF-alpha polymorphisms in subjects with irritable bowel syndrome in Mexico**. *Rev Esp Enferm Dig* 2013, **105**(7):392-399.

27. Romero-Valdovinos M, Gudino-Ramirez A, Reyes-Gordillo J, Martinez-Flores WA, Ramirez-Miranda ME, Maravilla P, Olivo-Diaz A: **Interleukin-8 and -10 gene polymorphisms in irritable bowel syndrome**. *Mol Biol Rep* 2012, **39**(9):8837-8843.

28. Zhang Y, Li Y, Hao Z, Li X, Bo P, Gong W: **Association of the Serotonin Receptor 3E Gene as a Functional Variant in the MicroRNA-510 Target Site with Diarrhea Predominant Irritable Bowel Syndrome in Chinese Women**. *J Neurogastroenterol Motil* 2016, **22**(2):272-281.

29. Camilleri M, Carlson P, McKinzie S, Zucchelli M, D'Amato M, Busciglio I, Burton D, Zinsmeister AR: **Genetic susceptibility to inflammation and colonic transit in lower functional gastrointestinal disorders: preliminary analysis**. *Neurogastroenterol Motil* 2011, **23**(10):398-935.

30. Zucchelli M, Camilleri M, Andreasson AN, Bresso F, Dlugosz A, Halfvarson J, Torkvist L, Schmidt PT, Karling P, Ohlsson B *et al*: **Association of TNFSF15 polymorphism with irritable bowel syndrome**. *Gut* 2011, **60**(12):1671-1677.

31. Choi YJ, Hwang SW, Kim N, Park JH, Oh JC, Lee DH: **Association Between SLC6A4 Serotonin Transporter Gene Lainked Polymorphic Region and ADRA2A -1291C>G and Irritable Bowel Syndrome in Korea**. *J Neurogastroenterol Motil* 2014, **20**(3):388-399.

32. Markoutsaki T, Karantanos T, Gazouli M, Anagnou NP, Ladas SD, Karamanolis DG: **Serotonin transporter and G protein beta 3 subunit gene polymorphisms in Greeks with irritable bowel syndrome**. *Dig Dis Sci* 2011, **56**(11):3276-3280.

33. Saito YA, Larson JJ, Atkinson EJ, Ryu E, Almazar AE, Petersen GM, Talley NJ: **The role of 5-HTT LPR and GNbeta3 825C>T polymorphisms and gene-environment interactions in irritable bowel syndrome (IBS)**. *Dig Dis Sci* 2012, **57**(10):2650-2657.

34. Wang Y, Wu Z, Qiao H, Zhang Y: **A genetic association study of single nucleotide polymorphisms in GNbeta3 and COMT in elderly patients with irritable bowel syndrome**. *Med Sci Monit* 2014, **20**:1246-1254.

35. Barkhordari E, Rezaei N, Ansaripour B, Larki P, Alighardashi M, Ahmadi-Ashtiani HR, Mahmoudi M, Keramati MR, Habibollahi P, Bashashati M *et al*: **Proinflammatory cytokine gene polymorphisms in irritable bowel syndrome**. *J Clin Immunol* 2010, **30**(1):74-79.

36. Karling P, Danielsson A, Wikgren M, Soderstrom I, Del-Favero J, Adolfsson R, Norrback KF: **The relationship between the val158met catechol-O-methyltransferase (COMT) polymorphism and irritable bowel syndrome**. *Plos One* 2011, **6**(3):e18035.

37. Tan L, Zhang D, Qiu X, Yang A, Ding S, Luo W: **Association between IL-2 T-384G polymorphisms and irritable bowel syndrome in Chinese Han population**. *Paractical Clinical Medicine* 2015(09):1-3.

38. Markoutsaki T, Karantanos T, Gazouli M, Anagnou NP, Karamanolis DG: **5-HT2A receptor gene polymorphisms and irritable bowel syndrome**. *J Clin Gastroenterol* 2011, **45**(6):514-517.

39. Pata C, Erdal E, Yazc K, Camdeviren H, Ozkaya M, Ulu O: **Association of the -1438 G/A and 102 T/C polymorphism of the 5-Ht2A receptor gene with irritable bowel syndrome 5-Ht2A gene polymorphism in irritable bowel syndrome**. *J Clin Gastroenterol* 2004, **38**(7):561-566.

40. Song YA, Park SY, Park YL, Chung CY, Lee GH, Cho DH, Park KJ, Cho SB, Lee WS, Joo YE: **Association between single nucleotide polymorphisms of the transient receptor potential vanilloid 1 (TRPV-1) gene and patients with irritable bowel syndrome in Korean populations**. *Acta Gastroenterol Belg* 2012, **75**(2):222-227.

41. Yuan J, Kang C, Wang M, Wang Q, Li P, Liu H, Hou Y, Su P, Yang F, Wei Y *et al*: **Association study of serotonin transporter SLC6A4 gene with Chinese Han irritable bowel syndrome**. *Plos One* 2014, **9**(1):e84414.

42. Sato N, Suzuki N, Sasaki A, Aizawa E, Obayashi T, Kanazawa M, Mizuno T, Kano M, Aoki M, Fukudo S: **Corticotropin-releasing hormone receptor 1 gene variants in irritable bowel syndrome**. *Plos One* 2012, **7**(9):e42450.

43. Kohen R, Jarrett ME, Cain KC, Jun SE, Navaja GP, Symonds S, Heitkemper MM: **The serotonin transporter polymorphism rs25531 is associated with irritable bowel syndrome**. *Dig Dis Sci* 2009, **54**(12):2663-2670.

44. Komuro H, Sato N, Sasaki A, Suzuki N, Kano M, Tanaka Y, Yamaguchi-Kabata Y, Kanazawa M, Warita H, Aoki M *et al*: **Corticotropin-Releasing Hormone Receptor 2 Gene Variants in Irritable Bowel Syndrome**. *Plos One* 2016, **11**(1):e147817.

45. Jun S, Kohen R, Cain KC, Jarrett ME, Heitkemper MM: **Associations of tryptophan hydroxylase gene polymorphisms with irritable bowel syndrome**. *Neurogastroenterol Motil* 2011, **23**(3):233-239, e116.

46. Shiotani A, Kusunoki H, Kimura Y, Ishii M, Imamura H, Tarumi K, Manabe N, Kamada T, Hata J, Haruma K: **S100A expression and interleukin-10 polymorphisms are associated with ulcerative colitis and diarrhea predominant irritable bowel syndrome**. *Dig Dis Sci* 2013, **58**(8):2314-2323.

47. Yeo A, Boyd P, Lumsden S, Saunders T, Handley A, Stubbins M, Knaggs A, Asquith S, Taylor I, Bahari B *et al*: **Association between a functional polymorphism in the serotonin transporter gene and diarrhoea predominant irritable bowel syndrome in women**. *Gut* 2004, **53**(10):1452-1458.

48. Grasberger H, Chang L, Shih W, Presson AP, Sayuk GS, Newberry RD, Karagiannides I, Pothoulakis C, Mayer E, Merchant JL: **Identification of a functional TPH1 polymorphism associated with irritable bowel syndrome bowel habit subtypes**. *Am J Gastroenterol* 2013, **108**(11):1766-1774.

49. Orand A, Naliboff B, Gadd M, Shih W, Ju T, Presson AP, Mayer EA, Chang L: **Corticotropin-releasing hormone receptor 1 (CRH-R1) polymorphisms are associated with irritable bowel syndrome and acoustic startle response**. *Psychoneuroendocrino* 2016, **73**:133-141.

50. Beyder A, Mazzone A, Strege PR, Tester DJ, Saito YA, Bernard CE, Enders FT, Ek WE, Schmidt PT, Dlugosz A *et al*: **Loss-of-Function of the Voltage-Gated Sodium Channel Na(V)1.5 (Channelopathies) in Patients With Irritable Bowel Syndrome**. *Gastroenterology* 2014, **146**(7):1659-1668.

**Additional Table2. Study characteristics analysis**

| Ref. | Country | Ethnicity | DNA  source | SNP detection methods | IBS  (M/F) | Dignostic criteria | IBS-C  (M/F) | IBS-D  (M/F) | IBS-A  (M/F) | IBS-M  (M/F) | Control  (M/F) | Control source | NOS | SNP rs. | HWE in controls |
| --- | --- | --- | --- | --- | --- | --- | --- | --- | --- | --- | --- | --- | --- | --- | --- |
| sVanderV et al;2005 | Netherland | Caucasian | Blood | PCR | 111  (35/76) | Rome II | 17 | 35 | 34 | / | 162 (101/61) | CC | 7 | IL10 rs1800896 | 0.71 |
|  |  |  |  |  |  |  |  |  |  |  |  |  |  | TNFα rs1800629 | 0.40 |
| ParkJM et al;2006 | Korea | Mongoloid | Blood | PCR | 190  (96/94) | Rome II | 54 | 97 | 39 | / | 437 (210/227) | CC | 6 | SLC6A45-HTTLPR | 0.20 |
| LiY et al;2007 | China | Mongoloid | Blood | PCR | 87  (32/55) | Rome II | 44 | 26 | 17 | / | 96 (39/57) | CC | 7 | SLC6A45-HTTLPR | 0.59 |
| KohenR et al;2009 | USA | Caucasian | Blood | PCR | 186  (27/159) | Rome II | / | / | / | / | 50 (15/35) | CC | 6 | SLC6A45-HTTLPR | 0.15 |
| SikanderA et al;2009 | India | Caucasian | Blood | PCR | 151  (113/38) | Rome II | 44 | 92 | / | 15 | 100 (64/36) | NM | 5 | SLC6A45-HTTLPR | 0.43 |
| Santhosh et al;2010 | India | Caucasian | Blood | PCR-SSP | 23 | Rome II | / | / | / | / | 20 | NM | 5 | IL10 rs1800871 | 0.66 |
|  |  |  |  |  |  |  |  |  |  |  |  |  |  | IL10 rs1800896 | 0.72 |
|  |  |  |  |  |  |  |  |  |  |  |  |  |  | TNFα rs1800629 | 0.52 |
| NieslerB et al;2010 | UK | Caucasian | Blood | PCR | 195  (34/161) | Rome II | 98 | 97 | / | / | 92 (32/60) | CC | 7 | SLC6A45-HTTLPR | 0.55 |
| Barkhordari et al;2010 | Iran | Iranian | Blood | PCR | 71  (22/49) | Rome **III** | / | / | / | / | 140 (70/70) | CC | 6 | IL10 rs1800871 | 0.91 |
|  |  |  |  |  |  |  |  |  |  |  |  |  |  | TNFα rs1800629 | 0.05 |
| Lee et al;2010 | Korea | Mongoloid | Buccal epithelial cell | PCR-RFLP | 94  (58/36) | Rome **III** | / | / | / | / | 88  (44/44) | CC | 6 | IL10 rs1800896 | 0.98 |
|  |  |  |  |  |  |  |  |  |  |  |  |  |  | TNFα rs1800629 | 0.38 |
| MarkoutsakiT et al;2011 | Greece | Caucasian | Blood | PCR-RFLP | 124  (30/94) | Rome **III** | / | / | / | / | 238  (96/142) | CC | 7 | GNβ3 rs5443 | 0.83 |
|  |  |  |  |  |  |  |  |  |  |  |  |  |  | SLC6A45-HTTLPR | 0.70 |
| Camilleri et al;2011 | USA | Caucasian, Hispanicor, Latino, Asian | Blood | PCR-TaqMan | 415 | Rome II | 156 | 175 | / | 84 | 231 | CC | 7 | IL23R rs11465804 | 0.61 |
|  |  |  |  |  |  |  |  |  |  |  |  |  |  | IL6 rs1800795 | 0.66 |
|  |  |  |  |  |  |  |  |  |  |  |  |  |  | TNFSF15 rs4263839 | 0.38 |
| JimenezG et al;2012 | Mexico | Mexican | Blood | PCR | 45  (9/36) | Rome **III** | 29 | 11 | / | 5 | 45  (15/30) | HC | 6 | IL6 rs1800795 | 0.44 |
|  |  |  |  |  |  |  |  |  |  |  |  |  |  | TNFα rs1800629 | 0.81 |
| KumarS et al;2012 | India | Caucasian | Blood | PCR | 150  (114/36) | Rome **III** | / | / | / | / | 252  (197/55) | NM | 5 | SLC6A45-HTTLPR | 0.31 |
| Zucchelli et al;2012 | Sweden | Caucasian | Blood | EMSA | 427 | Rome II | 104 | 144 | / | 179 | 900 | CC | 7 | IL23R rs11465804 | 0.63 |
|  |  |  |  |  |  |  |  |  |  |  |  |  |  | TNFSF15 rs4263839 | 0.54 |
| SaitoYA et al;2012 | USA | Caucasian | Blood | PCR | 385  (65/320) | Rome I/II | 40 | 102 | / | 125 | 262  (75/178) | CC | 7 | GNβ3 rs5443 | 1.00 |
|  |  |  |  |  |  |  |  |  |  |  |  |  |  | SLC6A45-HTTLPR | 0.98 |
| Swan et al;2012 | UK | Caucasian | Blood | PCR-TaqMan | 301 | Rome II | 122 | 179 | / | / | 179 | CC | 6 | IL10 rs1800896 | 0.82 |
|  |  |  |  |  |  |  |  |  |  |  |  |  |  | TNFα rs1800629 | 0.74 |
|  |  |  |  |  |  |  |  |  |  |  |  |  |  | TNFSF15 rs6478108 | 0.17 |
| Wouters et al;2013 | UK | Caucasian | Blood | PCR | 895 | Rome II | 325 | 430 | / | 140 | 639 | CC | 6 | IL23R rs11465804 | 0.89 |
|  |  |  |  |  |  |  |  |  |  |  |  |  |  | IL6 rs1800795 | 0.24 |
|  |  |  |  |  |  |  |  |  |  |  |  |  |  | TNFα rs1800629 | 0.19 |
|  |  |  |  |  |  |  |  |  |  |  |  |  |  | TNFSF15 rs4263839 | 0.67 |
|  |  |  |  |  |  |  |  |  |  |  |  |  |  | TNFSF15 rs6478108 | 0.85 |
| ColucciR et al;2013 | USA | Caucasian | Blood | PCR | 204  (45/159) | Rome **III** | 106 | 98 | / | / | 200  (46/154) | NM | 6 | SLC6A45-HTTLPR | 0.65 |
| FarjadianS et al;2013 | Iran | Caucasian | Blood | PCR | 50  (16/34) | Rome **III** | 15 | 25 | 10 | / | 100 | CC | 5 | SLC6A45-HTTLPR | 0.23 |
| SchmulsonM et al;2013 | Mexico | Mexican | Blood | PCR | 45  (11/34) | Rome II | 13(5/8) | 10(2/8) | / | 22(8/14) | 92  (36/56) | CC | 7 | IL10 rs1800896 | 0.14 |
| Shiotani et al;2013 | Japan | Mongoloid | Blood | PCR-RFLP | 50  (39/11) | Rome **III** | / | 50 | / | / | 50  (34/16) | HC | 6 | IL10 rs1800896 | 0.89 |
|  |  |  |  |  |  |  |  |  |  |  |  |  |  | SLC6A45-HTTLPR | 0.58 |
| RomeroV et al;2014 | Mexico | Mexican | Blood | PCR | 45  (7/38) | Rome **III** | / | / | / | / | 137  (41/96) | NM | 5 | IL10 rs1800871 | 0.92 |
| WangY et al;2014 | China | Mongoloid | Blood | PCR | 66  (56/10) | Rome **III** | 7 | 46 | / | 13 | 115  (89/26) | HC | 6 | GNβ3 rs5443 | 0.54 |
|  |  |  |  |  |  |  |  |  |  |  |  |  |  | COMT rs4680 |  |
| ChoiYJ et al;2014 | Korea | Mongoloid | Blood | PCR-TaqMan | 99  (38/61) | Rome **III** | 13 | 51 | / | 35 | 171  (85/86) | CC | 6 | GNβ3 rs5443 | 0.52 |
| CzogallaB et al;2015 | UK | Caucasian | Blood | PCR | 194  (36/158) | Rome II | 98 | 96 | / | / | 92  (32/60) | CC | 7 | IL10 rs1800871 | 0.51 |
|  |  |  |  |  |  |  |  |  |  |  |  |  |  | IL10 rs1800896 | 0.44 |
|  |  |  |  |  |  |  |  |  |  |  |  |  |  | IL23R rs11465804 | 0.70 |
|  |  |  |  |  |  |  |  |  |  |  |  |  |  | IL6 rs1800795 | 0.36 |
|  |  |  |  |  |  |  |  |  |  |  |  |  |  | TNFα rs1800629 | 0.96 |
|  |  |  |  |  |  |  |  |  |  |  |  |  |  | TNFSF15 rs4263839 | 0.14 |
|  |  |  |  |  |  |  |  |  |  |  |  |  |  | TNFSF15 rs6478108 | 0.14 |
| CzogallaB et al;2015 | USA | Caucasian | Sputum and/or Blood samples | PCR | 137  (27/110) | Rome **III** | 21 | 39 | / | 77 | 96  (46/50) | HC | 7 | IL10 rs1800871 | 0.56 |
|  |  |  |  |  |  |  |  |  |  |  |  |  |  | IL10 rs1800896 | 0.67 |
|  |  |  |  |  |  |  |  |  |  |  |  |  |  | IL23R rs11465804 | 0.37 |
|  |  |  |  |  |  |  |  |  |  |  |  |  |  | IL6 rs1800795 | 0.28 |
|  |  |  |  |  |  |  |  |  |  |  |  |  |  | TNFα rs1800629 | 0.62 |
|  |  |  |  |  |  |  |  |  |  |  |  |  |  | TNFSF15 rs4263839 | 0.25 |
|  |  |  |  |  |  |  |  |  |  |  |  |  |  | TNFSF15 rs6478108 | 0.75 |
| OrandA et al;2015 | USA | Caucasian, Asian, African American, Other/Mixed | salivary | PCR-TaqMan | 278 (68/209) | Rome **III** | 62 | 65 | 138 |  | 381  (102/279) | HC | 6 | COMT rs4680 |  |
| KarlingP et al;2011 | Sweden | Caucasian |  | PCR | 70 | Rome **III** |  |  |  |  | 867  (422/445) | CC | 5 | COMT rs4680 |  |
| KatsumataR et al;2018 | Japan | Mongoloid | Blood | PCR | 62 (40/22) | Rome **III** | / | / | / | / | 64 (42/22) | HC | 6 | SLC6A45-HTTLPR | 0.66 |

Note: IBS: irritable bowel syndrome; IBS-C: constipation predominant IBS; IBS-D: diarrhea predominant IBS; IBS-M: mixture of diarrhea and constipation IBS (Rome III); IBS-A: alternating of diarrhea and constipation (Rome II); M/F: male/female; NOS: Newcastle-Ottawa Quality Assessment Scale; HWE: Hardy-Weinberg equilibrium test;

CC: community control; HC: Hospital control; NM: not mentioned but with clear healthy control inclusion criteria;

Blood including "venous blood", "whole blood", "peripheral blood cells", "peripheral leukocytes";

Mongoloid including “Chinese”, “Japanese”, “Korean”;

EMSA: Electrophoretic Mobility Shift Assay;

PCR-SSP: Polymerase Chain Reaction- Sequence Specific Primers;

PCR-RFLP: Polymerase Chain Reaction-based restriction fragment length polymorphism;
